# **A** cross-sectional study to assess the epidemiological situation and associated risk factors of dengue fever; knowledge, attitudes, and practices about dengue prevention in Khyber Pakhtunkhwa Province, Pakistan

**DOI:** 10.3389/fpubh.2022.923277

**Published:** 2022-07-29

**Authors:** Jehangir Khan, Muhammad Adil, Gang Wang, Tsheten Tsheten, Dongjing Zhang, Wenjie Pan, Munir Ahmad Khan, Inayat ur Rehman, Xiaoying Zheng, Zhongdao Wu, Yu Wu

**Affiliations:** ^1^Department of Parasitology, Zhongshan School of Medicine, Sun Yat-sen University, Guangzhou, China; ^2^Key Laboratory of Tropical Disease Control of the Ministry of Education, Sun Yat-sen University-Michigan State University Joint Center of Vector Control for Tropical Disease, Zhongshan School of Medicine, Guangzhou, China; ^3^Chinese Atomic Energy Agency Center of Excellence on Nuclear Technology Applications for Insect Control, Sun Yat-sen University, Guangzhou, China; ^4^Department of Zoology, Abdul Wali Khan University Mardan, Mardan, Pakistan; ^5^Pakistan Bureau of Statistics, Islamabad, Pakistan; ^6^Department of Global Health, Research School of Population Health, College of Health and Medicine, Australian National University, Canberra, ACT, Australia; ^7^Royal Centre for Disease Control, Ministry of Health, Thimphu, Bhutan; ^8^Guangzhou SYSU Nuclear and Insect Biotechnology Co., Ltd., Guangzhou, China; ^9^Medical Unit, Khyber Teaching Hospital Peshawar, Peshawar, Pakistan; ^10^Department of Pharmacy, Abdul Wali Khan University Mardan, Mardan, Pakistan

**Keywords:** epidemiology, serology, DENV, SES, KAP

## Abstract

**Background:**

Dengue fever has been responsible for around 12 countrywide large outbreaks in Pakistan, resulting in 286,262 morbidities and 1,108 deaths. Khyber Pakhtunkhwa (KP) is the most recently impacted province. This study aimed to investigate the molecular, epidemiological, and potential elements that contribute to increasing dengue transmission patterns, and knowledge, attitude, and practice (KAP) toward dengue in KP province.

**Method:**

This cross-sectional community-based study was conducted (June-December, 2021) in two phases. Phase I involved the epidemiological (*n* = 5,242) and molecular analysis of DENV in 500 randomly collected blood samples of the 2021 dengue outbreak in KP. Phase II focused on assessing dengue-KAP levels in healthy communities (*n* = 14,745, aged >18 years), adopting a cross-sectional clustered multistage sampling in eight districts (dengue-hotspot vs. non-hotspot) of KP. Chi-square tests and logistic regression analysis were applied.

**Results:**

Peshawar district had the highest dengue cases (60.0%) associated with the predominant co-circulation of DENV-2 (45.8%) and DENV-3 (50.4%) serotypes. A rise in cases was reported in October (41.8%) followed by September (27.9%) and August (14.4%; *p* < 0.001). Males (63.7%, *p* < 0.001) and individuals aged 16–30 years (37.0%, *p* < 0.001) were highly affected. General workers (18.0%), families with a monthly income of 10,000–20,000 Pak rupees (50.5%), unmarried (71.0%), uneducated (31%), families with higher human density (>10 individuals per household), and those (29.0%) who faced power outages for more than 7/24 h were the most affected. Moreover, co-morbidities like renal failure and bronchial asthma were associated with disease severity. A community survey on KAP revealed that an average of 74, 60, and 43% of the participants demonstrated good knowledge, attitudes, and dengue preventive practices, respectively.

**Conclusion:**

Multiple poor socioeconomic elements are influencing dengue fever transmission in the province. Higher KAP levels may explain the low frequency of dengue in non-hotspot districts. Our study emphasizes the need for effective and long-term public health education, strengthened vector surveillance, and expanded laboratory capacity for better diagnosis and management of dengue cases to better predict the burden and seasonality of disease in the country.

## Introduction

Dengue fever (DF) is a vector-borne disease caused by one or more serotypes of dengue virus (DENV). Four DENV serotypes (DENV1-4) are currently identified to cause dengue in humans. Transmission occurs through the bites of *Aedes aegypti* and *Ae. albopictus*, a daytime biting mosquito species that reproduce in a variety of water-holding containers (both natural and manmade) and environments (rural, urban, and semi-urban) (1, 2).

Dengue has been constantly expanding (30-times increase over the past 50 years) to new areas and resurging in parts where it had recently been controlled (1). At present, dengue is widespread in over 129 countries and cases are rising constantly over the course of time: 0.51 million in 2000, 2.4 million in 2010, and 4.2 million in 2019. Asia represents about 70% of the world dengue disease burden (2). In particular, countries in the world health organization South-East Asia Region (WHO-SEAR) are severely affected with an estimated 1.3 billion people at risk, accounting for almost 52% of the world population at risk of contracting dengue virus (3). Dengue has significant negative financial and sociological consequences with a potential to considerably impede the development of economies, politics, and society (4).

Globally, transmission of dengue has been linked to a variety of social factors, including education level, household characteristics, overcrowding, water supply (i.e., poor sanitation and water-storing practices), electricity availability, vegetation cover, human behavior, and the abundance of *Aedes* mosquitoes (5, 6). To achieve effective disease control, communities should be empowered with right knowledge on dengue preventive strategies because human behaviors play a key role in facilitating dengue vectors (providing a favorable environment for breeding and blood meals) and disease transmission (5). Communities with higher socioeconomic status (SES) and a better understanding of dengue (in relation to knowledge, attitude, and prevention practices) have resulted in more successful disease control (5, 7). For example, in the Malaysian state of Selangor, human behavior has a significant impact on the spread and transmission of dengue (5, 7).

Dengue in Pakistan is also spreading at an alarming rate due to the wide range dispersion and adaptation of *Ae*. *aegypti* and *Ae. albopictus* (1). Dengue epidemics have been observed on a cyclical (2-to 3-year) basis, with an 8-fold increase in cases over the last decade. For example, a severe first dengue outbreak hit Peshawar (the country's third most populated city in KP) in 2017 that resulted in 24,938 cases and 70 deaths (8), followed by a second outbreak in 2021 that claimed over 10,000 cases and 10 deaths (9).

The public health intervention strategy for dengue relies heavily on massive insecticide spray in emergency and outbreak scenarios in Pakistan (10). A limited vector surveillance is also conducted including ovitraps, immature sampling for larva/pupae, and adult traps in various dengue prone districts (11, 12). Recently, an android based application named “Mosquito Alert Pakistan (MAP)” has been launched in Pakistan by the National Institute of Health (NIH), Islamabad. The application provides an early warning system for the risk of dengue transmission and other mosquito-borne diseases (13). Other control measures such as integrated vector management (IVM), with emphasis on habitat management, disposal of discarded tires, urban trash, and community awareness has been worked out very recently (14).

Previous studies have primarily focused on the epidemiology of dengue in the Peshawar district (8, 15). This is the first study to investigate the potential elements that contribute to increasing dengue transmission patterns and to assess the KAP on dengue in the KP province. Consequently, comprehensive epidemiological and molecular studies were carried out during the dengue outbreak to determine: (i) socio-demographic and clinical determinants of dengue fever; (ii) molecular analysis of DENV circulating; (iii) other societal variables like living standards, household type, power outages, impact of water storing practices, etc. that could be risk factors for dengue transmission; and (v) knowledge, attitude, and practice (KAP) about dengue prevention among general communities in dengue-hotspot and non-hotspot districts across the province. These findings will eventually provide a conceptual framework for building evidence-based, community-friendly, and long-term dengue preventive measures in Pakistan and elsewhere.

## Methodology

### Study settings

**Figure 1:**
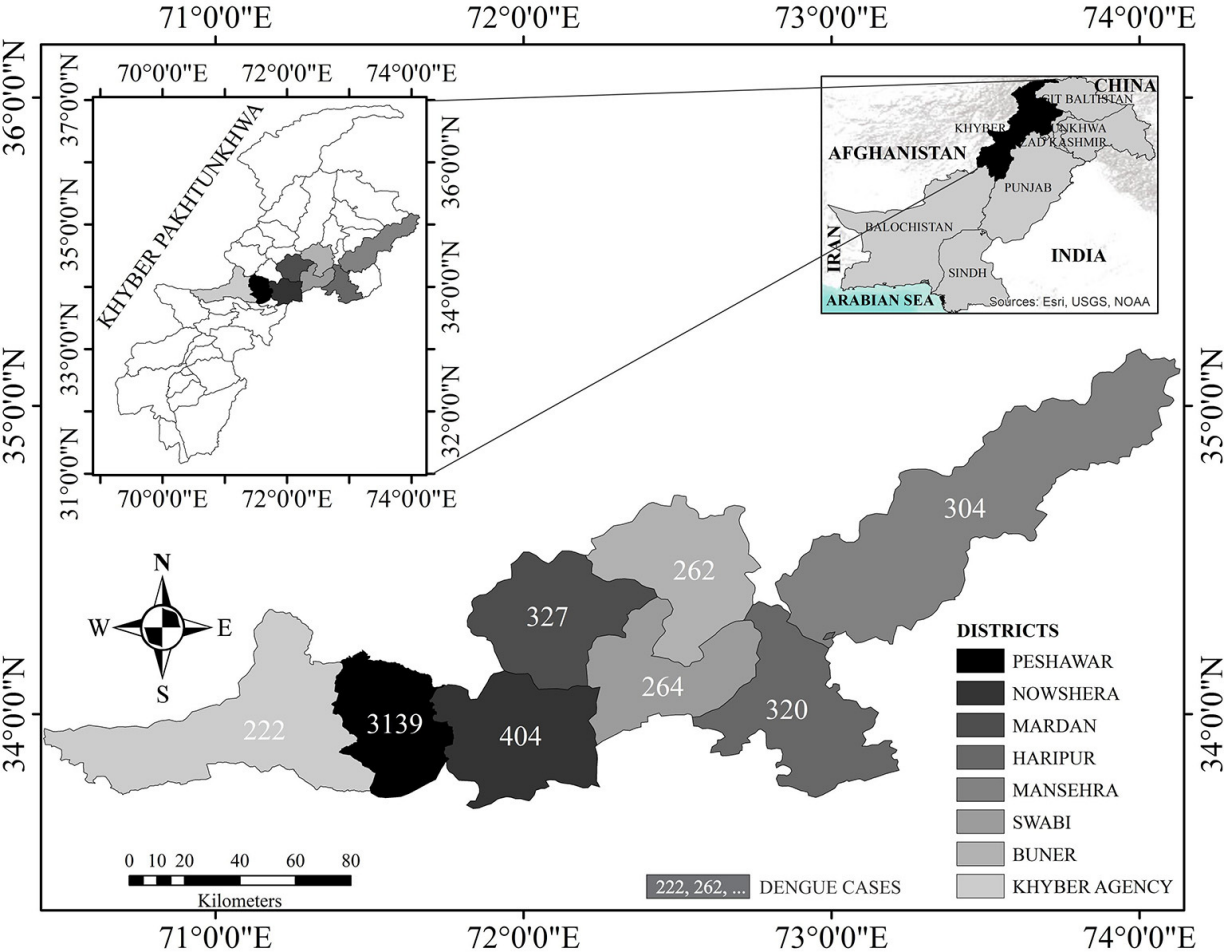
Map of Khyber Pakhtunkhwa province.

KP: 34.9526°N, 72.3311°E, formerly known as the North-West Frontier Province (NWFP), is the country's third-largest province by population and economy (Figure 1). It comprises of 17.9% (35.53 million) of Pakistan's overall population (2017 census) (16). Dengue transmission has increased in the province due to a variety of climatic settings, multiple tourist sites, rising urbanization, and increased travel and trade (17). Peshawar, the largest metropolitan city in KP with a population density of 1,612.5 per sq.km, has reported two massive dengue outbreaks in recent years (2017 and 2021). Peshawar is regarded as a hotspot for disease transmission (18). No dengue outbreaks had previously been documented in the other districts of the province except Swat, which observed an outbreak in 2013. KP can be divided into two zones: a dengue-endemic zone with only one district (Peshawar) and a non-endemic zone with seven districts (Mardan, Haripur, Nowshera, Swabi, Buner, Khyber, and Mansehra) (Figure 1). Thus, Peshawar is used as a reference for the rest (seven) of the surveyed districts according to set criteria (7).

### Study design

This cross-sectional community-based study took place during June-December, 2021 (dengue season) in two phases (independently) in dengue-hotspot and non-hotspot districts of KP province (mentioned above; Figure 1). The molecular (analytical), clinical, and epidemiological investigation of dengue patients (admitted in the dengue specified district headquarter hospital; DHQ) in each district throughout the outbreak was the focus of Phase I. Whereas, Phase II evaluated KAP on dengue among healthy communities, adopting a cross-sectional clustered multistage sampling in these eight districts. This study followed the STROBE principles for cross-sectional epidemiological studies (19) in terms of design, setting, analysis, and reporting.

### Sampling and sample size

In phase I, non-structural protein (NS1) or immunoglobulin IgG/IgM positive (active) patients across the province (having WHO defined dengue symptoms of dengue) (20) were considered for random blood sampling (4 ml of blood in EDTA tubes) during the initial days (0–5) of the disease (21) to isolate and characterize the DENV. The blood samples were immediately shifted to the dengue diagnostic laboratory in Peshawar and processed for further investigations (1). Following blood sampling, these patients were given a self-administered questionnaire adopted from previous studies (1, 22) (Supplementary Material 1) to fill out information about dengue disease clinical symptoms, duration, diagnostic results (i.e., platelet counts, etc.), and travel history to dengue endemic locations in the 5–10 days prior to the onset of symptoms, socioeconomic details like monthly income, household characteristics, power outages, other related information, and demographic details such as age and gender. The asset index developed by (23) was assumed to measure and classify participants' socioeconomic level (SES).

#### KAP measurements in phase II

**Figure 2:**
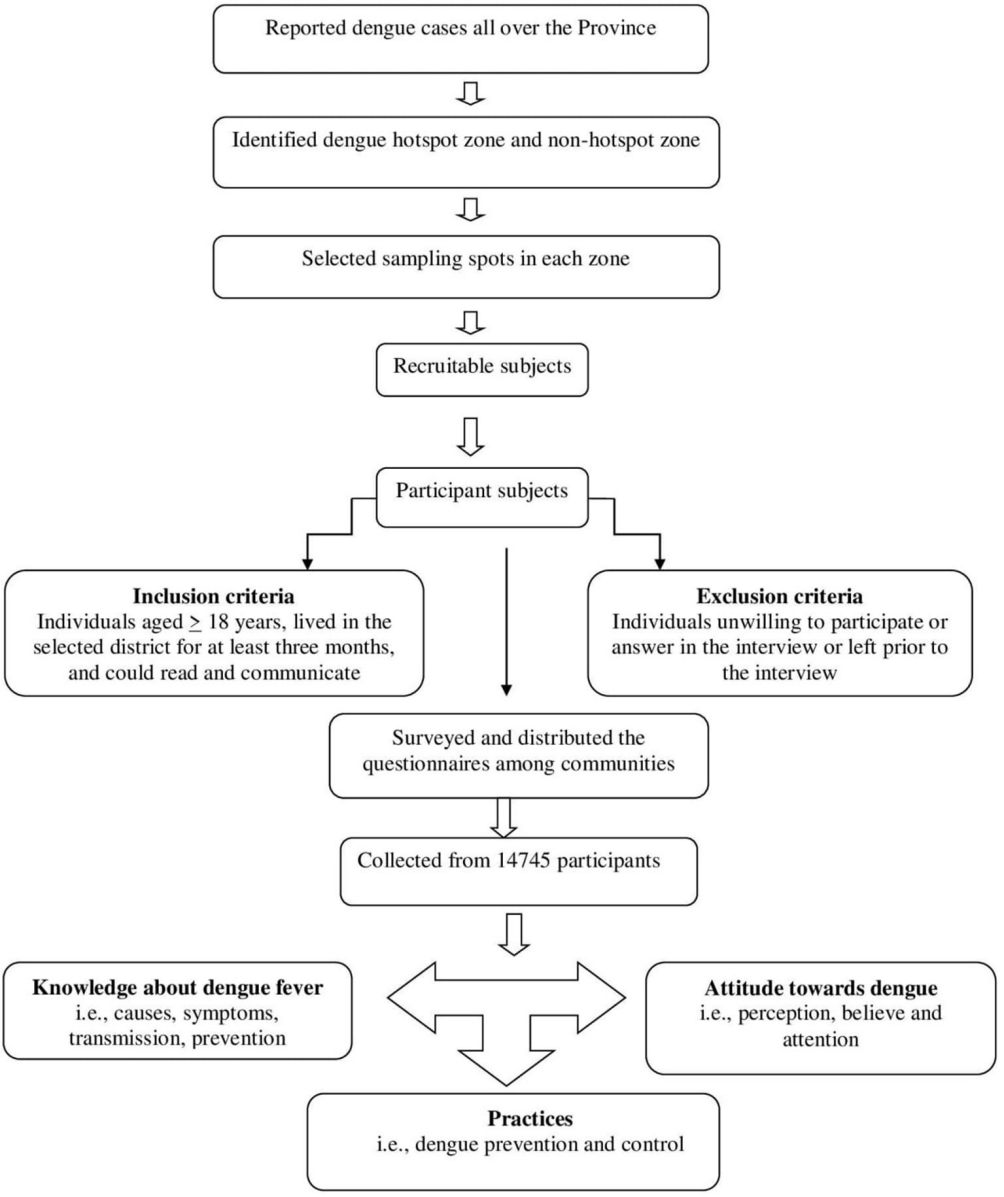
A conceptual framework diagram for a KAP-based (Phase II) study.

In phase II, we tested the hypothesis that good dengue KAP in healthy communities in non-dengue hotspot districts across the province could be the explanation for low dengue cases. Thus, an additional cross-sectional clustered multistage sampling approach (Figure 2) was undertaken to compare dengue-KAP levels in different communities (in hotspot and non-hotspot districts) and its subsequent impact on disease transmission according to Selvarajoo et al. (5). A dengue hotspot is defined as an area where a dengue outbreak has lasted longer than 30 days, whereas a non-hotspot is characterized as a place where there has been no dengue epidemic for longer than 30 days (7). A community's understanding of dengue, including its vectors and symptoms, is referred to as its knowledge. The methods people exhibit their knowledge and attitude *via* their actions are referred to as practices (5). For this, a questionnaire (Supplementary Material 2) was designed, in light of the 2009 WHO guidelines on dengue fever, which have been previously verified (5, 7). The questionnaire was comprised of two sections: (i) eight questions about knowledge and attitude (KA); and (ii) eight questions about dengue prevention practices (P). The study included participants who were over the age of 18, had lived in the selected district for at least 3 months, and could read and communicate (5, 7). Thus, a sample size of 14,745 participants was necessary to meet the study's objectives (24). Notably, the sample size from each district was based on population size together with the population density of the concerned district. The rationale for a large sample size was justified in order to ensure that enough respondents from across the province (eight districts) were included in the results, allowing the findings to be applied to a wide range of community contexts. Previous studies selected small sample size without taking into consideration the actual target population size. Here, in our study, the larger sample size is based on the previous dengue prevalence rates and the actual human population density in each target district. Therefore, the sample size was not uniform in each target district (Supplementary Material 3). The study participants were reached (weekdays during office hours) and interviews were requested after obtaining the written consent. Individuals who did not answer all of the questions, were unwilling to participate in the interview, or left prior to the interview were excluded. The data was subsequently entered (day wise) in the Microsoft Excel sheets (2010 version) and double-checked and validated for accuracy according to quality control protocols (21).

### Variables

Individual characteristics like age, gender, education level, socioeconomic status, and household characteristics, etc., were considered the independent variables in this study, while KAP scores (good vs. bad) and dengue incidence or DENV seropositivity (NS1/IgG) were considered as the dependent variable (25).

### Molecular identification of DENV serotypes

Reverse Transcription Polymerase Chain Reaction (RT-PCR) was used to identify the circulating DENV serotypes in the blood samples taken (randomly) from 500 NS1-positive hospitalized patients with dengue symptoms across the province (each district with 50 samples). The RNA extraction and PCR procedures were followed as described in recently published papers (1, 22). RNA was extracted using a Favorgine RNA extraction kit (CAT # FAVNKOO1-2) in line with the manufacturer's instructions. To identify the serotypes, the extracted RNA was processed using dengue virus type specific primers (TS1–TS4, plus D1) (26), in addition to positive (DENV-1, 2, 3, and 4) and negative controls. The amplified fragments were visualized in 2% agarose gels stained with Gel Red (Biotium Inc., USA).

### Statistical analysis and data interpretation/management

For the data (like epidemiological and socio-demographic and other associated risk factors etc.,) collected in Phases-I, we used the Chi-Square test, while for the data (KAP) collected in Phase-II, we adopted univariate and multivariate logistic regression analysis. Odds Ratios (ORs) were computed in the KAP survey to assess the magnitude of association between the outputs of a given category/district with a reference category/district (27). Given category is also known as the exposed category (non-hot spot area) while the one mentioned in reference category is considered as a control case/category (hot spot area). The statistical significance of the association was assessed using the 95% confidence interval. In this analysis Peshawar is chosen as a reference category due to highest dengue case load (hotspot). OR values less than or equal to one suggested similar attitude of the comparative non hotspot district to hotspot district, while a value >1 suggested that the odds of exposure among non-hot spot districts are greater than the odds of exposure among hot spot district. The predictors of each KAP domain were determined using logistic regression analysis. KAP responses as, “Yes” vs. “No,” were used as the outcome variables in the logistic regressions. Significant predictor factors from univariate analysis (*p* ≤ 0.25) were entered into the multivariate analysis. Confounding factors were explored by comparing the difference between the adjusted odds ratio (aOR) in multivariate analyses and the crude odds ratio (OR) in univariate analyses, of a particular predictor variable on the KAP domain. The correlation values among KAP scores and between KAP score and asset index were calculated using Spearman's rank correlation (rs) (28). SPSS and R softwares were used for the analysis. Ggplot in R was used to obtain boxplot which gave a five-point summary measure of the available data. The summary points are lower and upper quartiles, median, minimum and maximum values.

### Ethical considerations

The study and associated protocols were developed in accordance with national ethical legislation and as endorsed by the Ethic Board of the Zoology Faculty, Abdul Wali Khan University. In line with the latest version of the Declaration of Helsinki (29), all samples were obtained after the participants' written consent.

## Results

### Socio-demographic determinants of dengue fever

**Figure 3:**
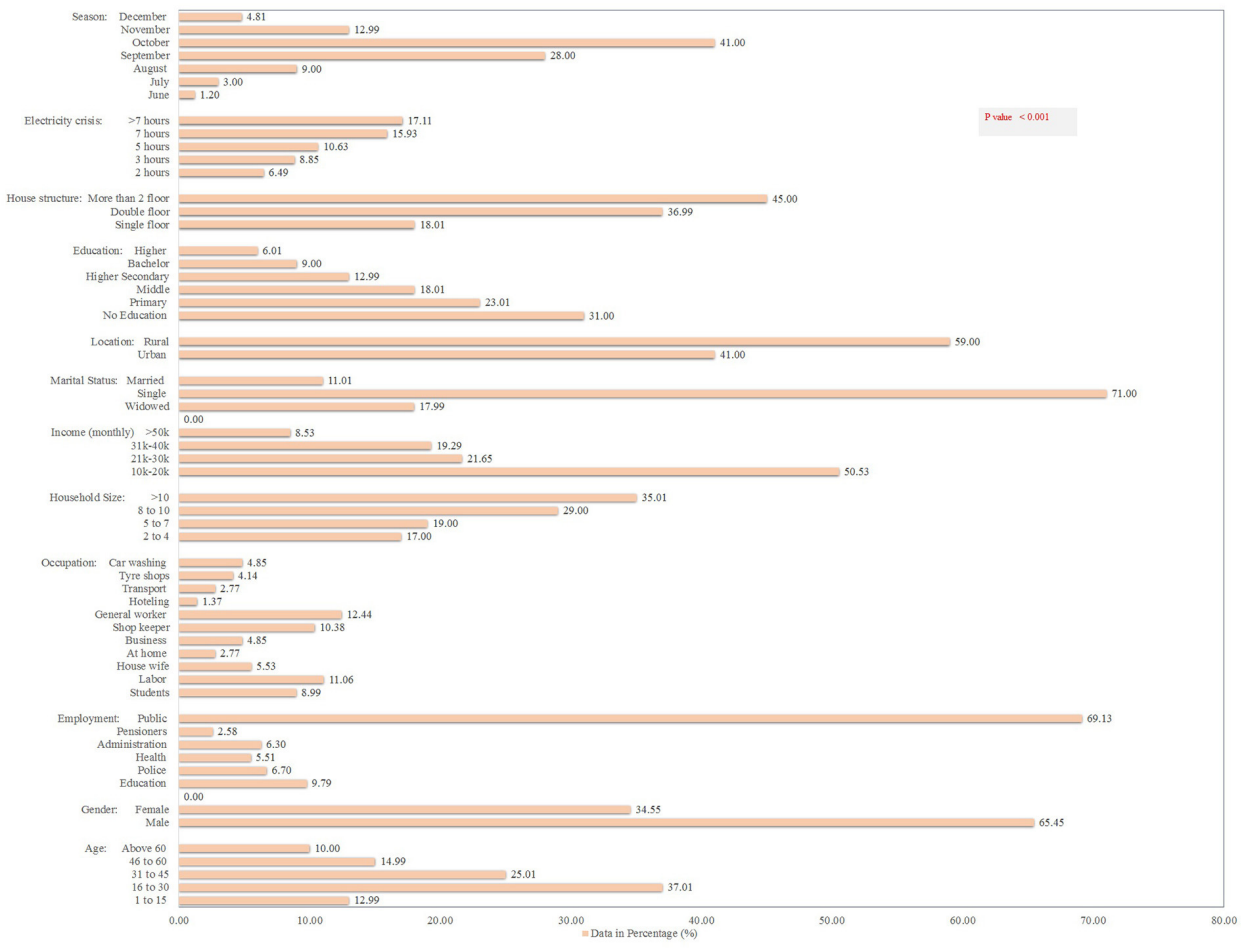
Socio-demographic determinants and other risk factors for dengue fever in the KP dengue outbreak (2021). Chi-square test was performed; *p* < 0.05 was considered statistically significant.

Figure 3 shows the demographic details of the 5,242 dengue patients reported in 2021, including their socioeconomic status (SES) and other associated risk factors. Dengue fever was more common in males (65%) and in people aged 16–30 years (37%) (*p* < 0.001). About 69% patients belonged to the general community (with little or no knowledge of dengue). In terms of occupation, general workers (working in various private organizations) had the highest dengue positive rates (18%), followed by the labor community (working on daily pay) (16%) (*p* < 0.000). Moreover, the majority (50.5%) of the patients had a monthly income of 10,000–20,000 Pakistan rupees (PKR) (*p* < 0.000). Single (unmarried) people had a higher rate of dengue fever (71%) than married people (11%). About 59% of the dengue patients belonged to rural areas. In terms of qualification, the highest frequency was identified in uneducated (31%) and primary-level educated (23%) communities (*p* < 0.000). About 29% of dengue fever was observed in areas with >7 h of power outages (*p* < 0.000). Surprisingly, families with a higher human density per household and communities living in multi-story houses had higher dengue prevalence rates of 35 and 45%, respectively (Figure 3).

#### Seasonal characteristics of dengue fever

The month-wise hospitalization of dengue patients revealed October with highest hospitalization rates (2,149; 41%) followed by September (1,468; 28%), whereas lowest rates were documented in June (63; 1.2%) and December (252; 4.8%; *p* < 0.001; Figure 3). Overall, the month and age wise data details of dengue occurrence in KP suggested significant association between the dengue occurrence and season (months).

#### Dengue highly affected districts in KP during 2021

Figure 1 demonstrates the number of dengue cases reported in each district. Among the reported confirmed dengue cases (5,242) and 10 deaths, Peshawar alone faced a load of 3,139 (60) cases, with three deaths only (*p* < 0.001). Nowshera and Mardan districts, adjacent to Peshawar, reported 404 (7.7%) and 327 (6.2%) incidences with one death each, respectively. Thus, Peshawar is considered the dengue hot-spot district (*p* < 0.001). Districts Khyber (4.2%) and Buner (5%), with one death each, reported a low number of dengue cases (*p* < 0.001). Surprisingly, the majority of these patients had visited dengue-endemic sites both within and outside the province (data not shown), showing that positive dengue cases were imported rather than locally acquired, which requires further investigations.

### Clinical characteristics of dengue fever

Table 1 shows the clinical characteristics of dengue fever. About 95% of cases were NS1+, and 15% of patients had blood platelets below 20,000. Among the dengue associated symptoms, 100% patients had fever and bone/muscles pain. About 97 and 93% of patients develop headaches and body aches, respectively. Abdominal pain was the second common symptom (84%) while bleeding was reported only in 8% patients. Dengue shock syndrome (DSS; 53.7%) and dengue hemorrhagic fever (DHF; 21%) were reported during the study (Table 1).

**Table 1:** Clinical characteristics of dengue fever during 2021.

**Clinical signs**	**Number (** ***n*** **)**		**Percentage (%)**		**95% C.I**	
			**Lower**		**Upper**
Fever	5,242		100	-		-
Body aches	4,875		93	92.31		93.69
Vomiting/ Nausea	2,883		55	53.65		56.34
Skin rashes	3,512		67	65.72		68.27
Bleeding	419		8	7.26		8.73
Haematemesis	1,572		30	28.75		31.23
Abdominal pain	4,403		84	83.00		84.99
Enlarged spleen	1,992		38	36.69		39.31
Bone pain	5,242		100	-		-
Muscles pain	5,242		100	-		-
Headache	5,084		97	96.52		97.45
**Hospital based dengue diagnosis results**				
NS1 +	4,980		95	94.41		95.59
IgM +	734		14	13.06		14.94
IgG +	367		7	6.31		7.69
PLT <100,000	2,359		45	43.66		46.35
PLT <50,000	2,097		40	38.68		41.33
PLT <20 000	786		15	14.03		15.96

**Dengue symptoms at the time of death**						
Symptom	Pulmonary edema with DHF	Renal failure with DHF	DSS	DHF	DF with bronchial asthma	Total
Number	1 (10%)	1 (10%)	3 (30%)	4 (40%)	1 (10%)	10

NS1, Non-structural proteins; PLT, Platelet count; IgM, Immunoglobulin M; IgG, Immunoglobulin G; DHF, Dengue hemorrhagic fever; DSS, Dengue shock syndrome.

### Distribution of DENV serotypes

**Figure 4:**
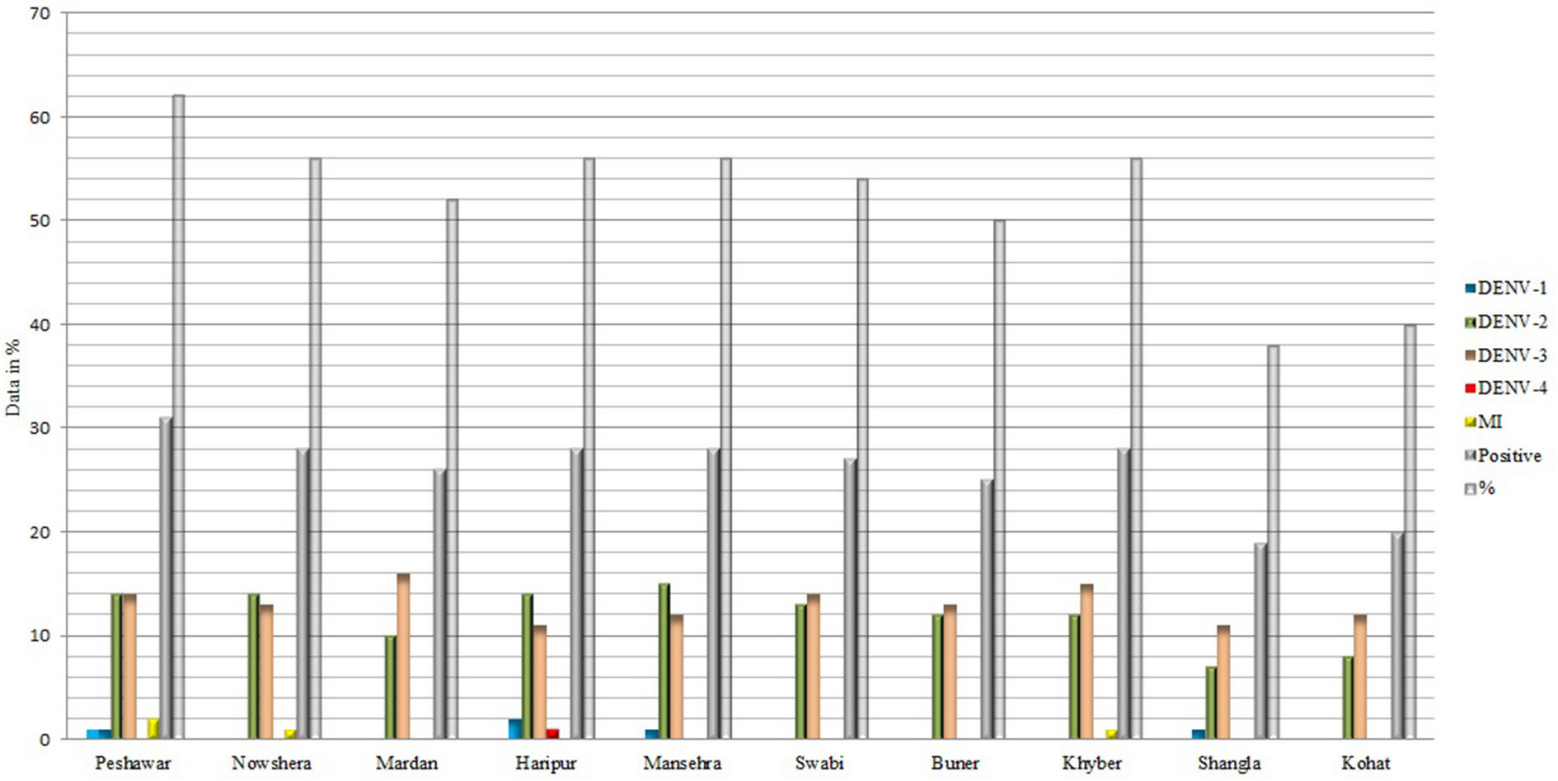
Molecular investigation of DENV among dengue patients across the province.

Out of the total blood samples tested (*n* = 500), only 260 (52%) samples were DENV positive (Figure 4). The dengue infection rates remained higher in district Peshawar (62%), whereas, Nowshera, Haripur, Mansehra, and Khyber had the similar infection rates (56%). Comparatively, the lowest infection rates were observed in districts Shangla (38%) and Kohat (40%). Regarding serotype distribution, DENV-3 (50.4%) and 2 (45.8%) were the predominant serotypes.

#### Socio-demographic characteristics of KAP-study population

**Figure 5:**
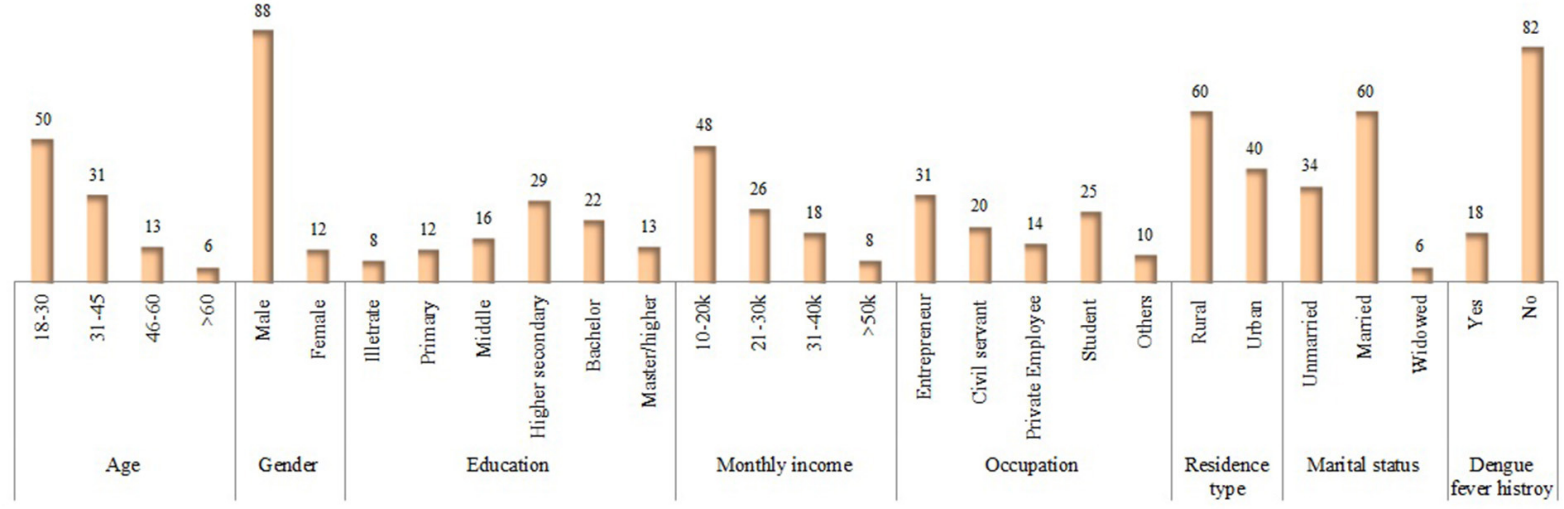
Socio-demographic characteristics of KAP-study participants in Phase II. Values are presented: *n* (%).

In Phase-II, the data was collected from 14,745 healthy participants from different eight districts across the province. Males constituted 88% of the total population, and half of the participants (50%) were 18–30 years old. Almost 29% of respondents had a higher secondary education certificate, and the majority (31%) was entrepreneurs (Figure 5). The majority of the residents (60%) belonged to rural areas, and nearly half of the participants (48%) had a monthly income of 10,000–20,000 Pakistan rupees (PKR). About 60% of the study subjects were married, and only 12% of the respondents had a history of dengue (Figure 5).

#### KAP levels among communities

##### Knowledge about dengue fever

We developed a series of logistic regression analyses to determine the factors independently associated with knowledge, attitude, and practice score for dengue prevention. Out of seven readily available parameters that had *p*-values < 0.25 in the univariate analysis, all factors were independently associated with knowledge, attitude and practice score for dengue. The findings showed that the odds of a knowledge score regarding dengue is 1.10 times higher for males as compared to females with (OR = 1.01, *p* < 0.001; Table 2). The odds of a knowledge score were 1.24 times higher in non-hot spot districts than in hot spot districts (OR = 1.24, *p* < 0.001). Regarding the education status, participants having education of middle and above had an odd knowledge score of 4.71 times higher than those with a lower education (OR = 4.71, *p* < 0.001). Similarly, the participants living in urban areas had an odd knowledge score that was 2.33 times higher as compared to rural areas (OR = 2.33, *p* < 0.001) as shown in Table 2. Similarly, those who had a history of dengue were more (OR = 1.4, *p* < 0.001) aware of the disease. The knowledge scores for married couples were 1.07 times higher than unmarried individuals (K; OR = 1.07, *p* < 0.05). However, there was no significant association between KAP, age, and SES.

**Table 2:** Predictors of KAP levels (good vs. poor) in univariate and multivariate analysis (*n* = 14,745).

**Predictors**	**Variable**	**Knowledge**						**Attitude**						**Practice**					
		**Univariate**			**Multivariate**			**Univariate**			**Multivariate**			**Univariate**			**Multivariate**		
		**O.R**	**95% CI**	***P***	**a.O.R**	**95% CI**	***P***	**O.R**	**95% CI**	***P***	**a.O.R**	**95% CI**	***P***	**O.R**	**95% CI**	***P***	**a.O.R**	**95% CI**	***P***
Gender	Male	1.15	0.74–1.29	0.001*	1.1	0.7–1.15	0.001*	2.6	2.3–2.75	0.001*	2.68	2.39–3.01	0.001*	2.15	1.95–2.34	0.020*	1.2	1.03–1.3	0.010*
	Female	Reference category																	
District	Hotspot	Reference category																	
	Non–hotspot	1.09	0.99–1.4	0.001*	1.24	1.09–1.42	0.001*	1.55	1.4–1.68	0.004*	0.829	0.72–0.94	0.004*	0.9	0.78–0.99	0.001*	0.85	0.8–0.9	0.001*
Marital status	Single	Reference category																	
	Married	2.02	1.85–2.22	0.23	1.07	0.7–1.6	0.05*	1.35	1.02–1.67	0.001*	1.29	1.05–1.61	0.001*	1.4	1.15–1.67	0.001*	1.13	0.95–1.34	0.001*
Education	Below Middle	Reference category																	
	Middle and above	1.09	0.99–1.2	0.001*	4.71	3.9–5.69	0.001*	2.5	2.1–2.86	0.001*	2.45	2.06–2.92	0.001*	2.4	2.12–2.7	0.001*	2.28	1.9–2.7	0.001*
Occupation	Private	Reference category																	
	Other	0.45	0.12–0.73	0.03*	0.223	0.19–0.27	0.001*	0.7	0.53–0.95	0.050*	0.9	0.85–1.05	0.001*	0.98	0.65–1.25	0.030*	0.95	0.6–1.2	0.040*
Residence	Urban	3.3	3.02–3.92	0.001*	2.33	1.99–2.73	0.001*	2.03	1.85–2.42	0.001*	1.5	1.1–1.9	0.001*	1.2	0.76–1.53	0.070*	1.29	0.96–1.6	0.001*
	Rural	Reference category																	
History of dengue	Yes	2.3	2.1–2.45	0.001*	1.365	1.2–1.6	0.001*	1.5	0.99–1.28	0.001*	1.1	0.95–1.15	0.001*	1.1	0.85–1.25	0.001*	1.397	1.24–1.6	0.001*
	No	Reference category																	
Age (years)	≤ 30	0.97	0.65–1.29	0.31				0.87	0.59–1.25	0.43				0.75	0.53–0.97	0.45			
	>30	Reference category																	
SES (PKR/month)	≤ 20,000	Reference category																	
	>20,000	1.3	0.97–1.63	0.45				1.45	1.10–1.70	0.53				1.7	1.35–2.05	0.37			

Univariate and multivariate logistic regression was performed, ^*^p-value < 0.05.

Overall, an average of 74% of participants demonstrated good knowledge of dengue and its symptoms (Supplementary Material 3). A district-wide analysis of dengue KAP levels revealed a considerable disparity in responses between communities in various districts (Supplementary Material 3, Figures 6A,B). All these districts were found to have statistically significant positive associations except the case of Peshawar vs. Mansehra (Supplementary Material 3). People in Peshawar, Mansehra, and Haripur districts were more aware of dengue fever, possibly as a result of a previous dengue outbreak in Peshawar and a few dengue cases reported in Mansehra and Haripur during 2004. It was also discovered that residents of district Khyber had a higher risk of exposure (odds for KAP) than residents of Peshawar.

**Figure 6:**
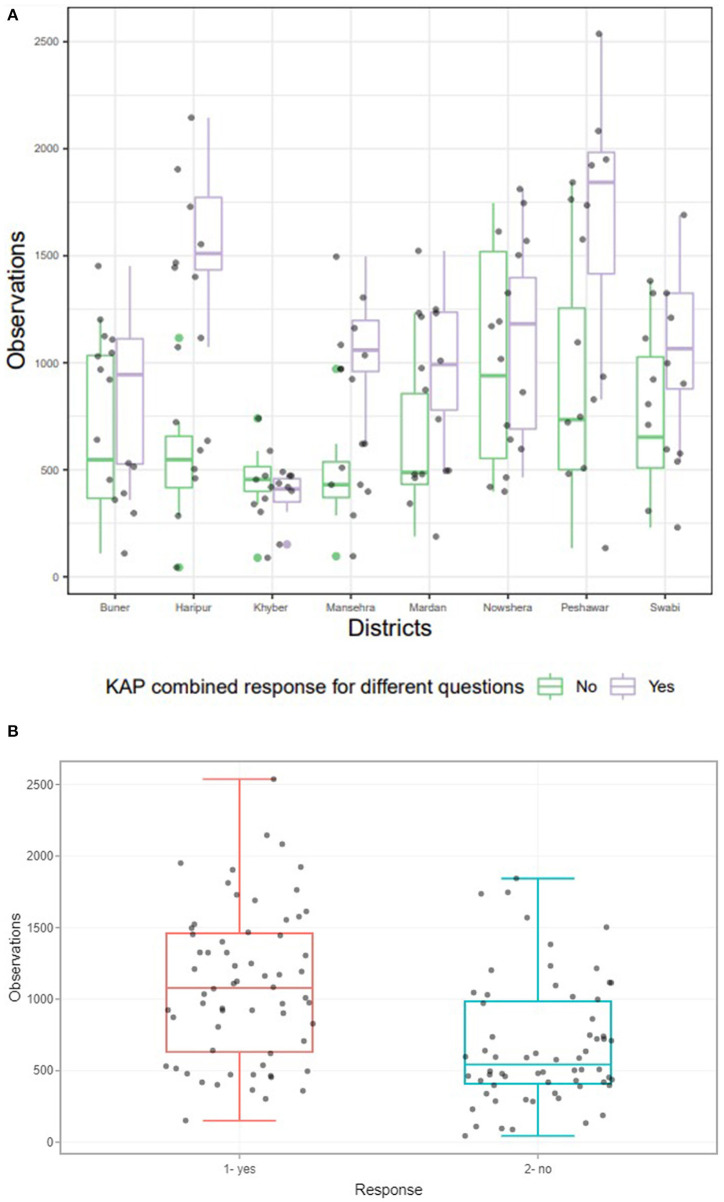
**(A)** District wise response of communities for questions mentioned in Supplementary Table 1. The above boxplot revealed significant relationship between the response of KAP for different questions for eight districts considered for study (*p* < 0.05). **(B)** Collective response of communities in the form of Yes and No for all districts to our questionnaires (Supplementary Table 1). Boxplot revealed significant association between collective responses of KAP questionnaire for different questions asked from eight districts considered for study. *P*-value was statistically significant at 5% level of significance, which was based on Chi Square test of independence.

##### Attitude toward dengue fever

Regarding the attitude score, the findings revealed that the odds of the attitude score regarding dengue was 2.68 times higher for males as compared to the attitude score regarding dengue in females (OR = 2.68, *p* < 0.001; Table 2). The odds of an attitude score were 0.829 times higher in non-hot spot districts than in hot spot districts (OR = 0.829, *p* < 0.004). In terms of education, participants having a middle and above had an odd attitude score that was 2.45 times higher than those having a lower middle (OR = 2.45, *p* < 0.001). Similarly, the participants living in urban areas have an average attitude score that is 1.50 times higher as compared to those in rural areas (OR = 1.50, *p* < 0.001) as shown in Table 2. The individuals with previous dengue history showed good attitude score (OR = 1.1, *p* < 0.001). The odd attitude scores for married subjects were 1.29 times higher than unmarried (OR = 1.29, *p* < 0.001). Overall, in terms of attitude, about 40% (average) of participants gave an unsatisfactory response (Supplementary Material 3).

#### Dengue prevention practices

Regarding the practice score, the findings demonstrated that the odds of the prevention practice score were 1.2 times higher for males as compared females (OR = 1.12, *p* < 0.010; Table 2). The odds of a practice score were 0.85 times higher in non-hotspot districts than in hotspot districts (OR = 0.85, *p* < 0.001). In terms of education, participants having a middle and above had an odd practice score that was 2.28 times higher than those having a lower middle (OR = 2.28, *p* < 0.001). Similarly, the participants living in urban areas have an odd practice score that is 1.29 times higher as compared to those in rural areas (OR = 1.29, *p* < 0.001) as shown in Table 2. Those who had dengue history demonstrated good practices (OR = 1.4, *p* < 0.001). The married subjects had 1.13 times practice odd practice score as compared to unmarried participants (OR = 1.13, *p* < 0.001).

Dengue preventative practices were indicated by roughly 43% of the respondents, with an emphasis on avoiding adult mosquito bites. Among the prevention practices, contact administration for fogging (29–68%) and the use of mosquito spray (48–63%) were most preferred strategies (Supplementary Material 3, Figures 6A,B). The widely used larval annihilation procedures include discarding stagnant water and scrub the container (~40%). In terms of opinions on different mosquito protection strategies, the most efficient option were full clothing (~37%) followed by chemical fogging (~25%) (Supplementary Material 3). The common approaches to self-protection were using mosquito repellent (~59%), followed by using mosquito nets (~53%). Interestingly, we observed two new methods, including burning dung cake (fumigation) and killing vector mosquito larvae with hot water (at 90–100°C). In general, our findings demonstrated that adult mosquito control approaches were prioritized over larvae/habitat destruction (source elimination). Finally, a strong correlation coefficient (*r* = 0.96) was found between the responses of those districts that had good knowledge and had taken adequate precautions to avoid dengue infection. However, a negative association (*r* = −0.287) was found for some districts (particularly Peshawar, Mardan, and Haripur) where people had good knowledge but were severely affected by dengue, possibly due to their failure to adopt the strict dengue prevention strategies.

## Discussion

This is the first study in the province to offer a comprehensive analysis of dengue epidemiology, risk factors, and the dengue-KAP. The current research (during sampling period) has documented a total of 5,242 confirmed dengue cases with 10 deaths across the province during 2021, with Peshawar being the hotspot reporting highest dengue cases (60%; Figure 1). Dengue fever is on the rise in the province, affecting people from all walks of life, particularly those with a poor standard of living. However, a substantial association between KAP levels and dengue control was found. Moreover, high education level, gender, marital status, dengue history, and living in the urban areas were all linked to good dengue-KAP (*p* < 0.001).

### Demographic and seasonal characteristics of dengue fever

Our result of dengue affecting more males than females is in congruent with the results obtained from Ahmad et al. (30) and other six Asian countries (31). However, the current finding is in contrast to research in South America that has reported either similar proportions of males and females or a larger ratio of female dengue patients (31). The higher prevalence of infection in male individuals may reflect their greater tendency to seek medical assistance and visit health facilities, resulting in more reporting, in contrast to females who choose traditional (at home) remedies for treatment (32). The other reasons might be attributed to a local cultural habit in which males often take off their shirts during the hot summer months and spend the entire night exposed to mosquito bites and disease transmission. Also, males are more exposed to mosquito bites (DENV-infected) during the daylight hours, either at work or on their way to and from work, at school, colleges, and universities (30, 33, 34). Secondly, females have a lower prevalence of dengue because they cover their bodies with full clothing (a cultural trait) and stay at home (with no free movement), limiting their exposure to mosquito bites and subsequent dengue transmission, an observation that agrees with (22, 35). The pattern of males being more infected with dengue is consistent across the country over time. For example, comparable outcomes were observed in a study conducted in two Pakistani metropolitan cities, Lahore and Multan (36). To target preventive strategies to lower the disease burden in the region, more studies are needed to determine the reasons for such sex-specific disparities.

The higher incidence of dengue in the economically productive age group in the current study (16–45) is consistent with other national and South Asian studies (1, 30, 32, 37–40). Contrary to our findings, others (22, 31) have reported higher dengue infection rates in the age group below 16 years, while others have reported higher incidence in older age groups (41). Such difference might be related to different local socio-demographic characteristics and warrants further research.

Our findings of higher dengue incidence in August, September and October might be related to higher temperatures and humidity that favors vector mosquito breeding. Similar finding was reported by (1, 12, 22, 32, 42). A severe power outage (particularly in August-October; Figure 3) exacerbates the dengue spread scenario by providing opportunities for mosquitoes to stay (on exposed human bodies), bite, and transmit DENV. Also, due to frequent power outages, local residents store water in various containers that serve as larval breeding habitats, resulting in an increase in mosquito population density. This promotes mosquito-human interaction, potentially resulting in DENV transmission, an observation similar to (44, 45). These observations are backed up by the fact that locations with >7/24 h power outages reported maximum (29%) dengue cases (Figure 3). This indicates that energy crises hastened the spread of dengue fever in the region.

### Socio-economic status and dengue fever transmission

Individuals with lower or no education and low monthly income had greater dengue occurrences than those with higher education and higher SES (Figure 3), similar to other Malaysian studies (13, 44–46). Firstly, individuals with a higher education and SES can have easy and frequent access to a variety of dengue-related information, allowing them to be aware of it and stay away from mosquito bites and disease transmission (25, 37, 47). Secondly, they can easily take advantage of all financial options to be safe, whereas a poor person or family cannot. For example, during hot seasons, when mosquito density is higher and human interaction is more likely, individuals with a better SES can stay safe (to avoid DENV infected mosquito bites) in air-conditioned rooms. Thirdly, they use alternative resources such as UPS (uninterrupted power supply) machines and electric generators (frequent power outage is a serious issue in Pakistan as discussed above). Poor families, on the other hand, are more exposed to mosquito bites (and disease transmission) due to lack of these resources, observations in line with (1, 22). Moreover, people with low SES and limited knowledge (about dengue) were less likely to take precautionary measures, including using mosquito nets, covering water containers, and changing water containers weekly, in line with others (18, 48–50). This could further explain the higher prevalence (59%) of dengue in rural areas (with limited resources) as compared to urban areas (49%; Figure 3). Likewise, residents of multi-story buildings and families with higher human densities were more vulnerable to dengue infection. This may suggest that vector-to-host ratios can explain epidemics in a given location, similar to studies (1, 22). Regarding employment, a large number of people infected with dengue belonged to education departments (513; 9.8%) and police department (*n* = 351; 6.7%; Figure 3). It's possible that these employees are more exposed to mosquito bites (infected with DENV) during work hours because they're in a crowded setting (as usually) in the workplace, which facilitates human-mosquito interactions and, subsequently, disease transmission, an observation in agreement with others (1, 22, 30).

### Household characteristics and dengue occurrence

Families with low human population densities (2–4 individuals per household) had a low dengue prevalence rate (17%) compared to higher densities (>10 individuals per household) which had a 35% prevalence rate (Figure 3). A household with more individuals offers more opportunities for female mosquitoes to have regular/frequent human-contacts, increasing the risk of virus transmission. Our hypothesis is supported by previous national and international studies (1, 12, 22, 47, 51). Interestingly, we noted higher dengue prevalence (71%) in unmarried (single) individuals than in married people (11%). This finding is in concordance with that of a Singapore study that linked living alone to a higher risk of dengue fever (52). This could be owing to a greater sense of obligation in married couples for their families than in single individuals. For example, married couples are more likely to use resources to provide a comfortable and safe environment (free of *Aedes* habitats) for their children at home and in the surroundings (50).

### Clinical symptoms and disease severity of dengue fever

WHO-defined clinical symptoms of dengue act as a significant tool in diagnosing and managing dengue fever (1). Rapid diagnosis of severe cases and effective clinical care are the mainstays of avoiding dengue-related case mortality (3). In our study, fever and bone/muscle pain was present in 100% of patients followed by headache (97%) and body aches (93%), with abdominal pain in 84% of patients and bleeding in only 8% of them (Table 1). Such milder symptoms are common for dengue infections across other countries in the Southeast Asia region (reviewed in Tsheten et al.) (3). Taken together, there is variation across studies regarding the severity of dengue. Vomiting and dehydration, as well as haematocrit with concurrent reduction in platelet count, were the most common (warning indicators) reasons for hospitalization in our study, which is consistent with studies from India (53), Thailand (54, 55), Bangladesh (56), and others (57). Here, severe dengue and case fatalities was associated with reduced platelet count, which is also reported in other studies (57–60). In our study, pulmonary infection, renal failure, and secondary infections were also associated with DHF and DSS, consistent with those of a study (3) that examined data from countries in the WHO-SEA region. This suggests that patients with renal and pulmonary infections should be given extra care in detecting the severity of their illness and receiving timely treatment. These characteristics, however, must be interpreted in conjunction with other lab tests (both recent and past). Furthermore, severe dengue was found to be a significant cause of morbidity and mortality in the country's older population in our study, in contrast to studies that found DHF and DSS to be a significant cause of hospitalization and death in children from Southeast Asia (61) and tropical regions (3, 62, 63). Thus, it recommends that elderly people and children should be given extra care during dengue epidemics and be prioritized for medical therapies to reduce the impact of dengue disease. Certain DENV serotypes, on the other hand, may have an impact on disease severity, such as secondary infection with DENV-1, 2, and 3 (64). Likewise, DENV-2 and DENV-4 have been highly associated with DSS, while DENV-3 and DENV-4 have been linked to DHF in the SEA region (65). Consistently, disease severity in our study can be attributed to the predominant serotypes 2 and 3, as very limited samples were diagnosed with DENV-1, DENV-4, and mix infection (Figure 4). It would be interesting if further detailed studies are conducted to explore the association between disease severity, DENV serotypes and age-related factors.

### Circulating DENV serotypes and dengue fever severity

DENV detection in human blood could be very effective in predicting impending epidemics. The current study observed 52% DENV positivity in blood (Figure 4). In Peshawar, however, higher DENV infection rates were found in blood samples. According to our findings, the predominant serotypes were DENV-2 (45.8%) and DENV-3 (50.4%), with DENV-1 (1.9%) and DENV-4 (0.4%) the least common serotypes (Figure 4), suggesting this outbreak is a continuation of the previous outbreak (2017). Our hypothesis is confirmed by recent studies conducted in the area (Peshawar) (66, 67). The epidemiological dynamics of dengue disease in a region are influenced by multiple serotypes (64). Previously, DENV-1, 2, 3, and 4 were identified as the primary cause of dengue epidemics in Pakistan (68–71). The severity of the disease in terms of high infection rates and pervasiveness in our study area is likely attributable to the serotypes DENV-2 and 3 and emergence of a new clade of DENV2 (cosmopolitan genotype IV: A1 lineage) (71) in 2017 dengue outbreak, an observation similar to other national and international studies (1, 3, 22, 51, 64, 69, 71, 72). Our claim is endorsed by (73), who mentioned that there are repeated extinctions of serotypes 2 and 3, which are replaced by new variants (more virulent) in the country, similar to others (65, 69, 72, 73). Different serotypes have been associated with different severity of dengue infections (64, 74). In the current study, both serotype 2 and 3 were associated with severe infections whereas previously DENV-2 was associated with almost 100 dengue infections in 2011 (69). Similar to our finding, Soo et al. (64) reported DENV-3 to have maximum percentage of primary infections (severity) in Southeast Asia (SEA) region whereas DENV-2 caused a larger percentage of severe dengue illnesses in non-SEA regions. In Thailand, all the severe primary infections were caused by serotype-1. Whereas, DENV-4 had the fewest cases in both SEA and non-SEA regions (64) and has been shown to be less immunogenic (75). Together, because DENV-2 and DENV-3 are more closely linked to dengue infection, serotype-specific antiviral medications may be customized to target these serotypes. Concurrent and secondary infections, on the other hand, were shown to be more severe than primary infections caused by any particular serotype (64). Thus, this demonstrates that while evaluating clinical outcomes and the severity of the illness, these serotypes should be given special attention. However, further studies (with increased sample size) determining serotype-specific disease severity and clinical symptoms will be fascinating and crucial to understanding epidemiological dynamics and evolutionary changes (genotypes/clades) in multi-strain disease systems in a given area.

### KAP-level

This is the first study to illustrate a possible link between KAP and dengue prevalence during a dengue outbreak in the province. Although good knowledge and attitude was observed among the communities, the dengue prevention practices were not much effective (Table 2 and Supplementary Material 3). However, communities with previous dengue exposure (i.e., Haripur, Masnsehra, and Peshawar) had a better level of awareness and attitudes toward dengue, with Khyber, Swabi, Buner, and Nowshera districts being significantly exposed to dengue infection based on low dengue-KAP (Supplementary Material 3). Fascinatingly, despite being aware of the disease's seriousness, having better SES, and having previously been exposed to dengue, residents of Peshawar, Mardan, Haripur, and Nowshera exhibited very limited interest in adopting strict preventive measures, which contradicts earlier findings (5, 7, 48–50). Despite the fact that males revealed higher KAP than females in our study (Table 2), the epidemiological investigation discovered a higher prevalence of dengue in males (Figure 3), possibly due to males' frequent exposure to mosquito bites and females' full clothing (a culturing trait) as discussed earlier. This requires further investigation. Taking together, this suggests that only knowledge is ineffective unless and until an individual's self-efficacy in adopting strict relevant precautions is strong, an observation similar to (7, 76). Consequently, the effective operation of dengue prevention measures requires good local government-community partnership.

Furthermore, very limited (~50%) knowledge regarding vector mosquitoes, their habits, and behavior was observed. For example, most communities confused *Aedes* mosquito species (which bite during dusk and dawn) with *Anopheles* and *Culex* species (night biters). Consequently, this increases the likelihood of *Aedes* mosquitoes breeding, biting, and transmitting DENV in human populations, similar to other national (37) and international studies (7, 48, 77). Taken together, this reveals a low level of KAP in the region when it comes to dengue prevention. Therefore, a nationwide survey is recommended to analyze the general public's knowledge and attitudes toward dengue fever, as well as any misunderstandings.

Likewise, among various options for dengue prevention, fogging and mosquito spray were the highly practiced methods (Supplementary Material 3). Self-protection methods included using mosquito repellent, bed nets, and full clothing. Vector control methods relied on discarding the larvae-infested water. Our findings on dengue prevention practices are in line with national (37, 78–80) and those of studies in India (81) and Nepal (82), but contradict with those of Malaysia (5, 7, 83, 84), Thailand (85), the Philippines (86), Yemen (87), and Jamaica (88), which revealed the participants had good and appropriate knowledge of dengue and dengue vectors. The disparity in outcomes could be due to differing governments' education and awareness initiatives in dengue-endemic countries, as indicated by the level of knowledge in communities. However, due to methodological discrepancies (for example, question type, community type, dissimilarity in respondents' background, scoring method, etc.), conclusions drawn from these studies must be interpreted with caution (5).

Notably, the current study observed that dengue prevention is predominantly focused on mosquito bite avoidance instead of mosquito eradication (through breeding site destruction and covering water containers, etc.), similar to other national report (37). It's crucial to note that discarding larvae-infested water near residences (as found in this study) is not a remedy for eradicating vector mosquitoes; rather, it allows mosquito populations to expand unnoticed in nature. Whereas, in the area, larvicidal spraying mostly targets homes, field sites for larval elimination are rarely inspected and treated. Subsequently, adult mosquitoes will reinvade homes in quest of a human host for blood, resulting in increased DENV transmission. Thus, antiseptics should be used to clean the larvae positive containers. The decreasing trend of dengue incidences in communities with adequate dengue knowledge (5, 17, 83) encourages measures to raise public awareness about dengue fever using a variety of information sharing approaches. The greater public awareness and motivation to eliminate mosquito breeding sites (*via* physical and chemical approaches), and the practice of using mosquito nets and complete clothing, will be critical in vector and disease management.

### Strengths and limitations of this study

Finally, our study has some limitations. First, some questions in the attitude domain may have a desirability bias. Second, since participants' KAP levels were only assessed once, the overall dynamic could shift over time. In contrast, one of our study's key strengths is that subjects (with a huge sample size) were recruited from all around the province, implying that the findings can be extended to a wide range of community settings. Determining a correlation between community-KAP-level and disease prevalence (based on epidemiological and molecular analysis) in each district further strengthens our study. The eligibility criteria further added to the quality of our results.

## Conclusion

We observed an increasing trend in DF caused by DENV serotypes 2 and 3, together with a high level of public attitude. However, there is still a profound ignorance and insensitivity regarding DF and proactive efforts for vector control. Individuals with low socioeconomic standing, and those with a lack of adequate information (dengue fever) and prevention measures, were particularly vulnerable to the disease. Moreover, the risk of dengue was higher in rural than in urban areas, largely explained by a lack of adequate resources and a poor level of KAP on dengue. Peshawar, Nowshera, and Mardan were among the most dengue-affected districts, with higher human population densities more frequently falling under the critical range. Our findings add to the body of knowledge that policymakers can use to develop guidelines aimed at addressing the root causes of the rising trend of dengue fever in KP province, in particular, and Pakistan in general. Moreover, it is strongly recommended that information concerning DF be disseminated by mass media, such as television, etc., in order to influence people's behavior and stop the impending outbreaks. The inclusion of DF preventive programmes into university and school curricula is highly recommended, particularly in places with a dearth of both high-quality healthcare resources and adequate health education.

## Data availability statement

The original contributions presented in the study are included in the article/Supplementary material, further inquiries can be directed to the corresponding authors.

## Ethics statement

The study and associated protocols were developed in accordance with National Ethical Legislation and as endorsed by the Ethic Board of the Zoology Faculty, Abdul Wali Khan University. In line with the latest version of the Declaration of Helsinki (29), all samples were obtained after the participants' written consent. The patients/participants provided their written informed consent to participate in this study.

## Author contributions

JK, ZW, XZ, and YW conceived and designed the experiments. JK, MA, GW, and IR analyzed the data. XZ, ZW, YW, DZ, GW, WP, and MA contributed reagents, materials, and analysis tools. TT critically revised the manuscript and provided suggestions and comments on the manuscript. MA formatted the figures. JK and MA interpreted and adjusted the figures in the manuscript. JK wrote the manuscript. All authors read and approved the final manuscript.

## Funding

This study was supported by the National Key R&D Program of China (no. 2020YFC1200100), National Natural Science Foundation of China (nos. 82002168 and 82072308), the 6th Nuclear Energy R&D Project (no. 20201192), and 111 project (no. B12003).

## Conflict of interest

Author WP was employed by Guangzhou SYSU Nuclear and Insect biotechnology Co., Ltd.

The remaining authors declare that the research was conducted in the absence of any commercial or financial relationships that could be construed as a potential conflict of interest.

## Publisher's note

All claims expressed in this article are solely those of the authors and do not necessarily represent those of their affiliated organizations, or those of the publisher, the editors and the reviewers. Any product that may be evaluated in this article, or claim that may be made by its manufacturer, is not guaranteed or endorsed by the publisher.

## Supplementary material

Supplementary material 1 Questionnaire 1.

Supplementary material 2 Questionnaire 2.

Supplementary material 3 The levels of knowledge, attitude, and practices (KAP) among people (*n* = 14,745) from various districts (KP) in 2021.
