# Supplementary material for: A cross-sectional study to assess the epidemiological situation and associated risk factors of dengue fever; knowledge, attitudes, and practices about dengue prevention in Khyber Pakhtunkhwa Province, Pakistan
Source: Front Public Health. 2022 Jul 29;10:923277. doi: 10.3389/fpubh.2022.923277 (PMC9372552; doi:10.3389/fpubh.2022.923277)
Supplement: Supplementary material 1 — Questionnaire 1. [file Data_Sheet_1.DOCX]

Respondent No.1

1. Name:________________, Gender________, Age________________, District_______,Home address____________ Place visited ___________, rural/urban___________.
2. Hospital or health care center_______, province________.
3. Date of dengue disease onset_________, duration of fever.
4. Symptoms, degree of fever: _________, body rashes_________, pain: muscle, bone _________, vomiting _______, bleeding ___________, headache_________, restlessness__________, platelet counts__________, and other symptoms___________________.
5. Dengue IgG/IGM or NS1__________.
6. Laboratory findings________________________________.
7. DHF symptoms _________________________________________________.
8. Education level_________________.
9. Monthly income __________________.
10. Married, single, widow_______.
11. Profession/employment/job_____________.
12. Individuals per household______________,
13. House type single/double or multiple storey_____.
14. Electricity availability or power outage_____________.
15. Water sources: tap water, spring, well or other resources__________.
16. Any others__________________________________.

# Summary
