# Supplementary material for: A cross-sectional study to assess the epidemiological situation and associated risk factors of dengue fever; knowledge, attitudes, and practices about dengue prevention in Khyber Pakhtunkhwa Province, Pakistan
Source: Front Public Health. 2022 Jul 29;10:923277. doi: 10.3389/fpubh.2022.923277 (PMC9372552; doi:10.3389/fpubh.2022.923277)
Supplement: Supplementary material 2 — Questionnaire 2. [file Data_Sheet_2.DOCX]

**Questionnaire 2**

Name:________________, Gender________, Age________________, District_______,

Home address____________ rural/urban___________.

**Knowledge and Attitude related questions**

1. Heard about dengue Yes/No
2. Dengue is caused via mosquito bit
3. Dengue mosquito lay eggs in clean water
4. Vector identification (white and dark patches)
5. Actions to prevent larval breeding in water
6. Action to control adult mosquito
7. Dengue is a community problem:Yes________ No____________ Don’t know______.
8. Opinion about dengue seriousness: Serious______, Moderate ________, Minor________.

**Dengue prevention practices related questions**

1. Contact administration for fogging
2. Personal use of insecticides
3. Physical search and destroy the breeding sites
4. Use mosquito spray

**Ways to protect yourself and family members from dengue infections**

1. Mosquito repellent
2. Bed nets
3. Destroy breeding sites
4. Insecticide

**Ways of larvae destruction**

1. Discard water with larvae
2. Use hot water to kill the larvae
3. Discard stagnant water and scrub the container
4. Discard water with larvae and wash it with antiseptic

**Opinion, which strategy is effective and do you adopt to prevent disease transmission**

1. Search & destroy mosquito breeding sites
2. Prevent from mosquito biting
3. Chemical fogging
4. Dung cake burning
5. Full clothing

**Summary:**

**_____________________________________________________________________________________________________________________________________________________________________________________________________________________.**
