# Supplementary material for: A cross-sectional study to assess the epidemiological situation and associated risk factors of dengue fever; knowledge, attitudes, and practices about dengue prevention in Khyber Pakhtunkhwa Province, Pakistan
Source: Front Public Health. 2022 Jul 29;10:923277. doi: 10.3389/fpubh.2022.923277 (PMC9372552; doi:10.3389/fpubh.2022.923277)
Supplement: Supplementary material 3 — The levels of knowledge, attitude, and practices (KAP) among people (n = 14,745) from various districts (KP) in 2021. [file Table_1.docx]

Table 1 shows the levels of knowledge, attitude, and practices (KAP) among people (n = 14745) from various districts (KP) in 2021.

| **Question** | **District** | | | | | | **Yes (%)** | | | | | | **No (%)** | | | | **Total** | | | **Odds Ratio** | | | | | **95% CI** | | | | | | | | **P value** | | |
| --- | --- | --- | --- | --- | --- | --- | --- | --- | --- | --- | --- | --- | --- | --- | --- | --- | --- | --- | --- | --- | --- | --- | --- | --- | --- | --- | --- | --- | --- | --- | --- | --- | --- | --- | --- |
| 1- Heard about dengue | **Peshawar** | | | | | | 2,537 (95) | | | | | | 134 (5) | | | | 2,671 | | | Reference | | | | | | | | | | | | | | | |
|  | **Mardan** | | | | | | 1,523 (89) | | | | | | 188 (11) | | | | 1,711 | | | 2.337 | | | | | 1.856 | | | | | 2.943 | | | | 0.000 | |
|  | **Khyber** | | | | | | 401 (45) | | | | | | 490 (55) | | | | 891 | | | 23.130 | | | | | 18.600 | | | | | 28.700 | | | | 0.000 | |
|  | **Haripur** | | | | | | 2,145 (98) | | | | | | 44 (2) | | | | 2,189 | | | 0.388 | | | | | 0.275 | | | | | 0.548 | | | | 0.000 | |
|  | **Mansehra** | | | | | | 1,496 (94) | | | | | | 96 (6) | | | | 1,592 | | | 1.210 | | | | | 0.928 | | | | | 1.590 | | | | 0.150 | |
|  | **Swabi** | | | | | | 1,690 (88) | | | | | | 230 (12) | | | | 1,920 | | | 2.570 | | | | | 2.060 | | | | | 3.200 | | | | 0.000 | |
|  | **Buner** | | | | | | 1,452 (95.8) | | | | | | 109 (4.2) | | | | 1,561 | | | 1.420 | | | | | 1.090 | | | | | 1.840 | | | | 0.008 | |
|  | **Nowshera** | | | | | | 1812 (82) | | | | | | 398 (18) | | | | 2,210 | | | 4.150 | | | | | 3.380 | | | | | 5.100 | | | | 0.000 | |
|  | **Total** | | | | | | | | | | | | | | | | **14,745** | | | Reference | | | | | | | | | | | | | | | |
| 2- Dengue is caused via mosquito bit | **Peshawar** | | | | | | 1,950 (73) | | | | | | 721 (27) | | | | 2,671 | | |  |  |  |  |  |  |  |  |  |  |  |  |  |  |  |  |
|  | **Mardan** | | | | | | 1,232 (72) | | | | | | 479 (28) | | | | 1,711 | | | 1.05 | | | | | 0.92 | | | 1.20 | | | | 0.725 | | | |
|  | **Khyber** | | | | | | 472 (53) | | | | | | 419 (47) | | | | 891 | | | 2.40 | | | | | 2.05 | | | 2.80 | | | | 0.000 | | | |
|  | **Haripur** | | | | | | 1,904 (87) | | | | | | 285 (13) | | | | 2,189 | | | 0.40 | | | | | 0.35 | | | 0.47 | | | | 0.000 | | | |
|  | **Mansehra** | | | | | | 1,305 (82) | | | | | | 287 (18) | | | | 1,592 | | | 0.59 | | | | | 0.51 | | | 0.69 | | | | 0.000 | | | |
|  | **Swabi** | | | | | | 1,325 (69) | | | | | | 595 (31) | | | | 1,920 | | | 1.21 | | | | | 1.06 | | | 1.38 | | | | 0.003 | | | |
|  | **Buner** | | | | | | 1,108 (71) | | | | | | 453 (29) | | | | 1,561 | | | 1.10 | | | | | 0.96 | | | 1.27 | | | | 0.155 | | | |
|  | **Nowshera** | | | | | | 1,613 (73) | | | | | | 597 (27) | | | | 2,210 | | | 1.00 | | | | | 0.88 | | | 1.13 | | | | 0.980 | | | |
|  | **Total** | | | | | | | | | | | | | | | | **14,745** | | | Reference | | | | | | | | | | | | | | | |
| 3- Dengue mosquito lay eggs in clean water | **Peshawar** | | | | | | 1,576 (59) | | | | | | 1,095 (41) | | | | 2,671 | | |  |  |  |  |  |  |  |  |  |  |  |  |  |  |  |  |
|  | **Mardan** | | | | | | 975 (57) | | | | | | 736 (43) | | | | 1,711 | | | 1.08 | | | | | 0.96 | | | 1.22 | | | | 0.180 | | | |
|  | **Khyber** | | | | | | 454 (51) | | | | | | 437 (49) | | | | 891 | | | 1.38 | | | | | 1.18 | | | 1.61 | | | | 0.000 | | | |
|  | **Haripur** | | | | | | 1,729 (79) | | | | | | 460 (21) | | | | 2,189 | | | 0.38 | | | | | 0.34 | | | 0.43 | | | | 0.000 | | | |
|  | **Mansehra** | | | | | | 1,162 (73) | | | | | | 430 (27) | | | | 1,592 | | | 0.53 | | | | | 0.46 | | | 0.60 | | | | 0.000 | | | |
|  | **Swabi** | | | | | | 1,210 (63) | | | | | | 710 (37) | | | | 1,920 | | | 0.84 | | | | | 0.74 | | | 0.95 | | | | 0.006 | | | |
|  | **Buner** | | | | | | 921 (59) | | | | | | 640 (41) | | | | 1,561 | | | 1.00 | | | | | 0.88 | | | 1.13 | | | | 0.990 | | | |
|  | **Nowshera** | | | | | | 1,193 (54) | | | | | | 1,017 (46) | | | | 2,210 | | | 1.22 | | | | | 1.09 | | | 1.37 | | | | 0.000 | | | |
|  | **Total** | | | | | | | | | | | | | | | | **14,745** | | | Reference | | | | | | | | | | | | | | | |
| 4- Vector identification (white and dark patches) | **Peshawar** | | | | | | 828 (31) | | | | | | 1,843 (69) | | | | 2,671 | | |  |  |  |  |  |  |  |  |  |  |  |  |  |  |  |  |
|  | **Mardan** | | | | | | 479 (28) | | | | | | 1232 (72) | | | | 1,711 | | | 1.15 | | | | | 1.01 | | | 1.32 | | | | 0.034 | | | |
|  | **Khyber** | | | | | | 151 (17) | | | | | | 740 (83) | | | | 891 | | | 2.20 | | | | | 1.81 | | | 2.67 | | | | 0.000 | | | |
|  | **Haripur** | | | | | | 1,554 (71) | | | | | | 635 (29) | | | | 2,189 | | | 0.18 | | | | | 0.16 | | | 0.20 | | | | 0.000 | | | |
|  | **Mansehra** | | | | | | 1,083 (68) | | | | | | 509 (32) | | | | 1,592 | | | 0.21 | | | | | 0.18 | | | 0.24 | | | | 0.000 | | | |
|  | **Swabi** | | | | | | 922 (48) | | | | | | 998 (52) | | | | 1,920 | | | 0.49 | | | | | 0.43 | | | 0.54 | | | | 0.000 | | | |
|  | **Buner** | | | | | | 531 (34) | | | | | | 1,030 (66) | | | | 1,561 | | | 0.87 | | | | | 0.76 | | | 0.99 | | | | 0.042 | | | |
|  | **Nowshera** | | | | | | 707 (32) | | | | | | 1,503 (68) | | | | 2,210 | | | 0.96 | | | | | 0.84 | | | 1.07 | | | | 0.450 | | | |
|  | **Total** | | | | | | | | | | | | | | | | **14,745** | | | Reference | | | | | | | | | | | | | | | |
| 5- Actions to prevent larval breeding in water | **Peshawar** | | | | | | 935 (35) | | | | | | 1,736 (65) | | | | 2,671 | | |  |  |  |  |  |  |  |  |  |  |  |  |  |  |  |  |
|  | **Mardan** | | | | | | 496 (29) | | | | | | 1215 (71) | | | | 1,711 | | | 1.32 | | | | | 1.15 | | | 1.50 | | | | 4.140 | | | |
|  | **Khyber** | | | | | | 303 (34) | | | | | | 588 (66) | | | | 891 | | | 1.04 | | | | | 0.89 | | | 1.22 | | | | 0.580 | | | |
|  | **Haripur** | | | | | | 1,073 (49) | | | | | | 1,116 (51) | | | | 2,189 | | | 0.56 | | | | | 0.50 | | | 0.63 | | | | 0.000 | | | |
|  | **Mansehra** | | | | | | 621 (39) | | | | | | 971 (61) | | | | 1,592 | | | 0.84 | | | | | 0.74 | | | 0.95 | | | | 0.009 | | | |
|  | **Swabi** | | | | | | 538 (28) | | | | | | 1,382 (72) | | | | 1,920 | | | 1.38 | | | | | 1.22 | | | 1.57 | | | | 0.000 | | | |
|  | **Buner** | | | | | | 359 (23) | | | | | | 1,202 (77) | | | | 1,561 | | | 1.80 | | | | | 1.56 | | | 2.07 | | | | 0.000 | | | |
|  | **Nowshera** | | | | | | 464 (21) | | | | | | 1,746 (79) | | | | 2,210 | | | 2.02 | | | | | 1.78 | | | 2.31 | | | | 0.000 | | | |
|  | **Total** | | | | | | | | | | | | | | | | **14,745** | | | Reference | | | | | | | | | | | | | | | |
| 6- Action to control adult mosquito | **Peshawar** | | | | | | 1,923 (72) | | | | | | 748 (28) | | | | 2,671 | | |  |  |  |  |  |  |  |  |  |  |  |  |  |  |  |  |
|  | **Mardan** | | | | | | 1,249 (73) | | | | | | 462 (27) | | | | 1,711 | | | 0.95 | | | | | 0.83 | | | 1.08 | | | | 0.460 | | | |
|  | **Khyber** | | | | | | 419 (47) | | | | | | 472 (53) | | | | 891 | | | 2.89 | | | | | 2.47 | | | 3.38 | | | | 0.000 | | | |
|  | **Haripur** | | | | | | 1,467 (67) | | | | | | 722 (33) | | | | 2,189 | | | 1.27 | | | | | 1.12 | | | 1.43 | | | | 0.000 | | | |
|  | **Mansehra** | | | | | | 971 (61) | | | | | | 621 (39) | | | | 1,592 | | | 1.64 | | | | | 1.44 | | | 1.87 | | | | 0.000 | | | |
|  | **Swabi** | | | | | | 806 (42) | | | | | | 1,114 (58) | | | | 1,920 | | | 3.55 | | | | | 3.14 | | | 4.02 | | | | 0.000 | | | |
|  | **Buner** | | | | | | 515 (33) | | | | | | 1,046 (67) | | | | 1,561 | | | 5.22 | | | | | 4.56 | | | 5.97 | | | | 0.000 | | | |
|  | **Nowshera** | | | | | | 641 (29) | | | | | | 1,569 (71) | | | | 2,210 | | | 6.29 | | | | | 5.55 | | | 7.13 | | | | 0.000 | | | |
|  | **Total** | | | | | | | | | | | | | | | | **14,745** | | |  |  |  |  |  |  |  |  |  |  |  |  |  |  |  |  |
| **7- Dengue is a community problem** | | | | | | | | | | | | | | | | | | | | | | | | | | | | | | | | | | | |
| **Peshawar** | | | **Yes** | | | | | **No** | | | | **Don’t know** | | | | | | **Total** | | | Reference | | | | | | | | | | | | | | |
|  |  |  | 2,084(78) | | | | | 507 (19) | | | | 80 (3) | | | | | | 2,671 | | |  |  |  |  |  |  |  |  |  |  |  |  |  |  |  |
| **Mardan** | | | 1,010(59) | | | | | 496 (29) | | | | 205 (12) | | | | | | 1,711 | | | 2.01 | | | | 1.74 | | | | 2.33 | | | 0.000 | | | |
| **Khyber** | | | 472 (53) | | | | | 339 (38) | | | | 80 (9) | | | | | | 891 | | | 2.95 | | | | 2.48 | | | | 3.49 | | | 0.000 | | | |
| **Haripur** | | | 1,446 (66) | | | | | 503 (23) | | | | 240 (11) | | | | | | 2,189 | | | 1.43 | | | | 1.24 | | | | 1.64 | | | 0.000 | | | |
| **Mansehra** | | | 1,035 (65) | | | | | 430 (27) | | | | 127 (8) | | | | | | 1,592 | | | 1.70 | | | | 1.47 | | | | 1.98 | | | 0.000 | | | |
| **Swabi** | | | 1,325 (69) | | | | | 307 (16) | | | | 288 (15) | | | | | | 1,920 | | | 0.95 | | | | 0.81 | | | | 1.11 | | | 0.540 | | | |
| **Buner** | | | 969 (62) | | | | | 390 (25) | | | | 202 (13) | | | | | | 1,561 | | | 1.65 | | | | 1.42 | | | | 1.92 | | | 0.000 | | | |
| **Nowshera** | | | 1,326 (60) | | | | | 420 (19) | | | | 464 (21) | | | | | | 2,210 | | | 1.30 | | | | 1.12 | | | | 1.50 | | | 0.000 | | | |
|  |  |  | **Total** | | | | | | | | | | | | | | | **14,745** | | |  |  |  |  |  |  |  |  |  |  |  |  |  |  |  |
| **8- Opinion about dengue seriousness*** | | | | | | | | | | | | | | | | | | | | | | | | | | | | | | | | | | | |
| **Peshawar** | | | | **Serious** | | | | **Moderate** | | | | | **Minor** | | | | | **Total** | | | Reference | | | | | | | | | | | | | | |
|  |  |  |  | 1,763(66) | | | | 481 (18) | | | | | 427 (16) | | | | | 2,671 | | |  |  |  |  |  |  |  |  |  |  |  |  |  |  |  |
| **Mardan** | | | | 873 (51) | | | | 342 (20) | | | | | 496 (29) | | | | | 1,711 | | | 1.43 | | | | 1.22 | | | | 1.68 | | | 0.000 | | | |
| **Khyber** | | | | 366 (41) | | | | 89 (10) | | | | | 436 (49) | | | | | 891 | | | 0.89 | | | | 0.69 | | | | 1.15 | | | 0.871 | | | |
| **Haripur** | | | | 1,401(64) | | | | 591 (27) | | | | | 197 (9) | | | | | 2,189 | | | 3.95 | | | | 3.48 | | | | 4.47 | | | 0.000 | | | |
| **Mansehra** | | | | 924 (58) | | | | 398 (25) | | | | | 270 (17) | | | | | 1,592 | | | 1.58 | | | | 1.35 | | | | 1.85 | | | 0.000 | | | |
| **Swabi** | | | | 903 (47) | | | | 576 (30) | | | | | 441 (23) | | | | | 1,920 | | | 2.34 | | | | 2.02 | | | | 2.70 | | | 0.000 | | | |
| **Buner** | | | | 1,124(72) | | | | 297 (19) | | | | | 140 (9) | | | | | 1,561 | | | 0.96 | | | | 0.82 | | | | 1.14 | | | 0.700 | | | |
| **Nowshera** | | | | 1,172(53) | | | | 862 (39) | | | | | 176 (8) | | | | | 2,210 | | | 2.69 | | | | 2.36 | | | | 3.08 | | | 0.000 | | | |
|  |  |  |  | **Total** | | | | | | | | | | | | | | **14,745** | | |  |  |  |  |  |  |  |  |  |  |  |  |  |  |  |
| **Question** | | | | | | **District** | | | | **Yes (%)** | | | | | **No (%)** | | | | | **Odds ratio** | | | | | **95% CI** | | | | | | | **P value** | | | |
| 1- Contact administration for fogging | | | | | | **Peshawar** | | | | 1,522 (57) | | | | | 1,149 (43) | | | | | Reference | | | | | | | | | | | | | | | |
|  |  |  |  |  |  | **Mardan** | | | | 890 (52) | | | | | 821 (48) | | | | | 1.22 | | | | | 1.08 | | | 1.38 | | | | 0.0010 | | | |
|  |  |  |  |  |  | **Khyber** | | | | 258 (29) | | | | | 633 (71) | | | | | 3.25 | | | | | 2.76 | | | 3.83 | | | | 0.0001 | | | |
|  |  |  |  |  |  | **Haripur** | | | | 1,357 (62) | | | | | 832 (38) | | | | | 0.81 | | | | | 0.72 | | | 0.91 | | | | 0.0004 | | | |
|  |  |  |  |  |  | **Mansehra** | | | | 1,083 (68) | | | | | 509 (32) | | | | | 0.62 | | | | | 0.54 | | | 0.71 | | | | 0.000 | | | |
|  |  |  |  |  |  | **Swabi** | | | | 922 (48) | | | | | 998 (52) | | | | | 1.43 | | | | | 1.27 | | | 1.61 | | | | 0.000 | | | |
|  |  |  |  |  |  | **Buner** | | | | 827 (53) | | | | | 734 (48) | | | | | 1.18 | | | | | 1.04 | | | 1.33 | | | | 0.012 | | | |
|  |  |  |  |  |  | **Nowshera** | | | | 1,083 (49) | | | | | 1,127 (51) | | | | | 1.38 | | | | | 1.23 | | | 1.54 | | | | 0.000 | | | |
| 2- Personal use of insecticides | | | | | | **Peshawar** | | | | 1,549 (58) | | | | | 1,122 (42) | | | | | Reference | | | | | | | | | | | | | | | |
|  |  |  |  |  |  | **Mardan** | | | | 907 (53) | | | | | 804 (47) | | | | | 1.22 | | | | | 1.08 | | | 1.38 | | | | 0.001 | | | |
|  |  |  |  |  |  | **Khyber** | | | | 454 (51) | | | | | 437 (49) | | | | | 1.32 | | | | | 1.14 | | | 1.54 | | | | 0.000 | | | |
|  |  |  |  |  |  | **Haripur** | | | | 1,204 (55) | | | | | 985 (45) | | | | | 1.13 | | | | | 1.01 | | | 1.26 | | | | 0.036 | | | |
|  |  |  |  |  |  | **Mansehra** | | | | 971 (61) | | | | | 621 (39) | | | | | 0.88 | | | | | 0.78 | | | 1.00 | | | | 0.054 | | | |
|  |  |  |  |  |  | **Swabi** | | | | 998 (52) | | | | | 922 (48) | | | | | 1.27 | | | | | 1.13 | | | 1.43 | | | | 0.000 | | | |
|  |  |  |  |  |  | **Buner** | | | | 796 (51) | | | | | 765 (49) | | | | | 1.32 | | | | | 1.17 | | | 1.50 | | | | 0.000 | | | |
|  |  |  |  |  |  | **Nowshera** | | | | 1,061 (48) | | | | | 1,149 (52) | | | | | 1.50 | | | | | 1.33 | | | 1.67 | | | | 0.000 | | | |
| 3- Physical search and destroy the breeding sites | | | | | | **Peshawar** | | | | 1,122 (42) | | | | | 1,549 (58) | | | | | Reference | | | | | | | | | | | | | | | |
|  |  |  |  |  |  | **Mardan** | | | | 667 (39) | | | | | 1044 (61) | | | | | 1.13 | | | | | 1.00 | | | 1.28 | | | | 0.047 | | | |
|  |  |  |  |  |  | **Khyber** | | | | 196 (22) | | | | | 695 (78) | | | | | 2.57 | | | | | 2.15 | | | 3.06 | | | | 0.000 | | | |
|  |  |  |  |  |  | **Haripur** | | | | 657 (30) | | | | | 1,532 (70) | | | | | 1.68 | | | | | 1.50 | | | 1.90 | | | | 0.000 | | | |
|  |  |  |  |  |  | **Mansehra** | | | | 732 (46) | | | | | 860 (54) | | | | | 0.85 | | | | | 0.75 | | | 0.96 | | | | 0.011 | | | |
|  |  |  |  |  |  | **Swabi** | | | | 749 (39) | | | | | 1,171 (61) | | | | | 1.13 | | | | | 1.00 | | | 1.27 | | | | 0.042 | | | |
|  |  |  |  |  |  | **Buner** | | | | 390 (25) | | | | | 1,171 (75) | | | | | 2.17 | | | | | 1.90 | | | 2.50 | | | | 0.000 | | | |
|  |  |  |  |  |  | **Nowshera** | | | | 530 (24) | | | | | 1,680 (76) | | | | | 2.30 | | | | | 2.02 | | | 2.60 | | | | 0.000 | | | |
| 4- Use mosquito spray | | | | | | **Peshawar** | | | | 1,683 (63) | | | | | 988 (37) | | | | | Reference | | | | | | | | | | | | | | | |
|  |  |  |  |  |  | **Mardan** | | | | 924 (54) | | | | | 787 (46) | | | | | 1.45 | | | | | 1.28 | | | 1.64 | | | | 0.000 | | | |
|  |  |  |  |  |  | **Khyber** | | | | 517 (58) | | | | | 374 (42) | | | | | 1.23 | | | | | 1.05 | | | 1.43 | | | | 0.008 | | | |
|  |  |  |  |  |  | **Haripur** | | | | 1,116 (51) | | | | | 1073 (49) | | | | | 1.63 | | | | | 1.46 | | | 1.83 | | | | 0.000 | | | |
|  |  |  |  |  |  | **Mansehra** | | | | 939 (59) | | | | | 653 (41) | | | | | 1.18 | | | | | 1.04 | | | 1.34 | | | | 0.009 | | | |
|  |  |  |  |  |  | **Swabi** | | | | 998 (52) | | | | | 922 (48) | | | | | 1.57 | | | | | 1.39 | | | 1.78 | | | | 0.000 | | | |
|  |  |  |  |  |  | **Buner** | | | | 765 (49) | | | | | 796 (51) | | | | | 1.77 | | | | | 1.56 | | | 2.01 | | | | 0.000 | | | |
|  |  |  |  |  |  | **Nowshera** | | | | 1,061 (48) | | | | | 1,149 (52) | | | | | 1.84 | | | | | 1.64 | | | 2.06 | | | | 0.000 | | | |
| **Ways to protect yourself and family members from dengue infections** | | | | | | | | | | | | | | | | | | | | | | | | | | | | | | | | | | | |
| 5- Mosquito repellent | | | | | | | **Peshawar** | | | | 1,442 (54) | | | | 1,229 (46) | | | | | Reference | | | | | | | | | | | | | | | |
|  |  |  |  |  |  |  | **Mardan** | | | | 873 (51) | | | | 838 (49) | | | | | 1.13 | | | | | 0.99 | | | 1.27 | | | | 0.0550 | | | |
|  |  |  |  |  |  |  | **Khyber** | | | | 428 (48) | | | | 463 (52) | | | | | 1.27 | | | | | 1.09 | | | 1.48 | | | | 0.0020 | | | |
|  |  |  |  |  |  |  | **Haripur** | | | | 1,204 (55) | | | | 985 (45) | | | | | 0.96 | | | | | 0.86 | | | 1.07 | | | | 0.4790 | | | |
|  |  |  |  |  |  |  | **Mansehra** | | | | 939 (59) | | | | 653 (41) | | | | | 0.82 | | | | | 0.72 | | | 0.93 | | | | 0.002 | | | |
|  |  |  |  |  |  |  | **Swabi** | | | | 826 (43) | | | | 1,094 (57) | | | | | 1.55 | | | | | 1.38 | | | 1.75 | | | | 0.000 | | | |
|  |  |  |  |  |  |  | **Buner** | | | | 765 (49) | | | | 796 (51) | | | | | 1.22 | | | | | 1.07 | | | 1.38 | | | | 0.002 | | | |
|  |  |  |  |  |  |  | **Nowshera** | | | | 1,149 (52) | | | | 1,061 (48) | | | | | 1.08 | | | | | 0.96 | | | 1.21 | | | | 0.160 | | | |
| 6- Mosquito nets | | | | | | | **Peshawar** | | | | 1,095 (41) | | | | 1,576 (59) | | | | | Reference | | | | | | | | | | | | | | | |
|  |  |  |  |  |  |  | **Mardan** | | | | 770 (45) | | | | 941 (55) | | | | | 0.85 | | | | | 0.75 | | | 0.95 | | | | 0.008 | | | |
|  |  |  |  |  |  |  | **Khyber** | | | | 472 (53) | | | | 419 (47) | | | | | 0.61 | | | | | 0.53 | | | 0.72 | | | | 0.000 | | | |
|  |  |  |  |  |  |  | **Haripur** | | | | 832 (38) | | | | 1357 (62) | | | | | 1.13 | | | | | 1.01 | | | 1.27 | | | | 0.034 | | | |
|  |  |  |  |  |  |  | **Mansehra** | | | | 716 (45) | | | | 876 (55) | | | | | 0.85 | | | | | 0.75 | | | 0.96 | | | | 0.011 | | | |
|  |  |  |  |  |  |  | **Swabi** | | | | 614 (32) | | | | 1306 (68) | | | | | 1.47 | | | | | 1.30 | | | 1.67 | | | | 0.000 | | | |
|  |  |  |  |  |  |  | **Buner** | | | | 812 (52) | | | | 749 (48) | | | | | 0.64 | | | | | 0.56 | | | 0.72 | | | | 0.000 | | | |
|  |  |  |  |  |  |  | **Nowshera** | | | | 840 (38) | | | | 1370 (62) | | | | | 1.13 | | | | | 1.00 | | | 1.27 | | | | 0.034 | | | |
| 7- Destroy breeding sites | | | | | | | **Peshawar** | | | | 588 (22) | | | | 2,083 (78) | | | | | Reference | | | | | | | | | | | | | | | |
|  |  |  |  |  |  |  | **Mardan** | | | | 342 (20) | | | | 1,369 (80) | | | | | 1.13 | | | | | 0.97 | | | 1.31 | | | | 0.109 | | | |
|  |  |  |  |  |  |  | **Khyber** | | | | 125 (14) | | | | 766 (86) | | | | | 1.73 | | | | | 1.40 | | | 2.13 | | | | 0.000 | | | |
|  |  |  |  |  |  |  | **Haripur** | | | | 263 (12) | | | | 1,926 (88) | | | | | 2.06 | | | | | 1.77 | | | 2.42 | | | | 0.000 | | | |
|  |  |  |  |  |  |  | **Mansehra** | | | | 398 (25) | | | | 1,194 (75) | | | | | 0.85 | | | | | 0.73 | | | 0.98 | | | | 0.025 | | | |
|  |  |  |  |  |  |  | **Swabi** | | | | 461 (24) | | | | 1,459 (76) | | | | | 0.89 | | | | | 0.78 | | | 1.03 | | | | 0.112 | | | |
|  |  |  |  |  |  |  | **Buner** | | | | 297 (19) | | | | 1,264 (81) | | | | | 1.20 | | | | | 10.02 | | | 1.40 | | | | 0.021 | | | |
|  |  |  |  |  |  |  | **Nowshera** | | | | 398 (18) | | | | 1,812 (82) | | | | | 1.29 | | | | | 1.11 | | | 1.48 | | | | 0.000 | | | |
| 8- Insecticide | | | | | | | **Peshawar** | | | | 962 (36) | | | | 1,709 (64) | | | | | Reference | | | | | | | | | | | | | | | |
|  |  |  |  |  |  |  | **Mardan** | | | | 462 (27) | | | | 1,249 (73) | | | | | 1.52 | | | | | 1.33 | | | 1.73 | | | | 0.000 | | | |
|  |  |  |  |  |  |  | **Khyber** | | | | 98 (11) | | | | 793 (89) | | | | | 4.55 | | | | | 3.63 | | | 5.70 | | | | 0.000 | | | |
|  |  |  |  |  |  |  | **Haripur** | | | | 438 (20) | | | | 1,751 (80) | | | | | 2.25 | | | | | 1.97 | | | 2.56 | | | | 0.000 | | | |
|  |  |  |  |  |  |  | **Mansehra** | | | | 350 (22) | | | | 1,242 (78) | | | | | 1.99 | | | | | 1.73 | | | 2.30 | | | | 0.000 | | | |
|  |  |  |  |  |  |  | **Swabi** | | | | 365 (19) | | | | 1,555 (81) | | | | | 2.40 | | | | | 2.08 | | | 2.75 | | | | 0.000 | | | |
|  |  |  |  |  |  |  | **Buner** | | | | 281 (18) | | | | 1280 (82) | | | | | 2.56 | | | | | 2.20 | | | 2.98 | | | | 0.000 | | | |
|  |  |  |  |  |  |  | **Nowshera** | | | | 663 (30) | | | | 1547 (70) | | | | | 1.31 | | | | | 1.16 | | | 1.48 | | | | 0.000 | | | |
| ***1-Ways of larvae destruction (source eradication)** | | | | | | | | | | | | | | | | | | | | | | | | | | | | | | | | | | | |
| Peshawar | | | Discard water with larvae  N (%) | | | | | | | Use hot water to kill the larvae  N (%) | | | | | | Discard stagnant water and scrub the container N (%) | | | | | | | | Discard water with larvae and wash it with antiseptic N (%) | | | | | | | | | | | |
|  |  |  | 935 (35) | | | | | | | 641 (24) | | | | | | 721 (27) | | | | | | | | 374 (14) | | | | | | | | | | | |
| Mardan | | | 548 (32) | | | | | | | 479 (28) | | | | | | 479 (28) | | | | | | | | 205 (12) | | | | | | | | | | | |
| Khyber | | | 250 (28) | | | | | | | 276 (31) | | | | | | 285 (32) | | | | | | | | 80 (9) | | | | | | | | | | | |
| Haripure | | | 722 (33) | | | | | | | 416 (19) | | | | | | 591 (27) | | | | | | | | 460 (21) | | | | | | | | | | | |
| Mansehra | | | 318 (20) | | | | | | | 239 (15) | | | | | | 637 (40) | | | | | | | | 398 (25) | | | | | | | | | | | |
| Swabi | | | 557 (29) | | | | | | | 460 (24) | | | | | | 614 (32) | | | | | | | | 289 (15) | | | | | | | | | | | |
| Buner | | | 375 (24) | | | | | | | 328 (21) | | | | | | 671 (43) | | | | | | | | 187 (12) | | | | | | | | | | | |
| Nowshera | | | 796 (36) | | | | | | | 552 (25) | | | | | | 641 (29) | | | | | | | | 221 (10) | | | | | | | | | | | |
| ***2- Opinion: which strategy is effective and do you adopt to prevent disease transmission** | | | | | | | | | | | | | | | | | | | | | | | | | | | | | | | | | | | |
| Peshawar | | | Search & destroy mosquito breeding sites N (%) | | | | | | | | | Prevent from mosquito biting N (%) | | | | | Chemical fogging  N (%) | | | | | | Dung cake burning  N (%) | | | | Full clothing  N (%) | | | | | | | | |
|  |  |  | 240 (9) | | | | | | | | | 561 (21) | | | | | 748 (28) | | | | | | 454 (17) | | | | 668 (25) | | | | | | | | |
| Mardan | | | 188 (11) | | | | | | | | | 308 (18) | | | | | 325 (19) | | | | | | 325 (19) | | | | 565 (33) | | | | | | | | |
| Khyber | | | 125 (14) | | | | | | | | | 169 (19) | | | | | 98 (11) | | | | | | 169 (19) | | | | 330 (37) | | | | | | | | |
| Haripure | | | 285 (13) | | | | | | | | | 438 (20) | | | | | 504 (23) | | | | | | 262 (12) | | | | 700 (32) | | | | | | | | |
| Mansehra | | | 350 (22) | | | | | | | | | 334 (21) | | | | | 302 (19) | | | | | | 175 (11) | | | | 431 (27) | | | | | | | | |
| Swabi | | | 288 (15) | | | | | | | | | 326 (17) | | | | | 269 (14) | | | | | | 346 (18) | | | | 691 (36) | | | | | | | | |
| Buner | | | 250 (16) | | | | | | | | | 297 (19) | | | | | 203 (13) | | | | | | 265 (17) | | | | 546 (35) | | | | | | | | |
| Nowshera | | | 221 (10) | | | | | | | | | 464 (21) | | | | | 553 (25) | | | | | | 287 (13) | | | | 685 (31) | | | | | | | | |

Univariate analysis of KAP in 8 districts of KPK.

***Statistics:** Q1 (χ^2^ = 622.5, DF = 21, P<0.000), Q2 (χ^2^ = 492.6, DF = 21, P<0.000). P values are calculated based on chi square test of independence. ***** For odd ratios calculation, seriousness and moderate classifications were considered.
